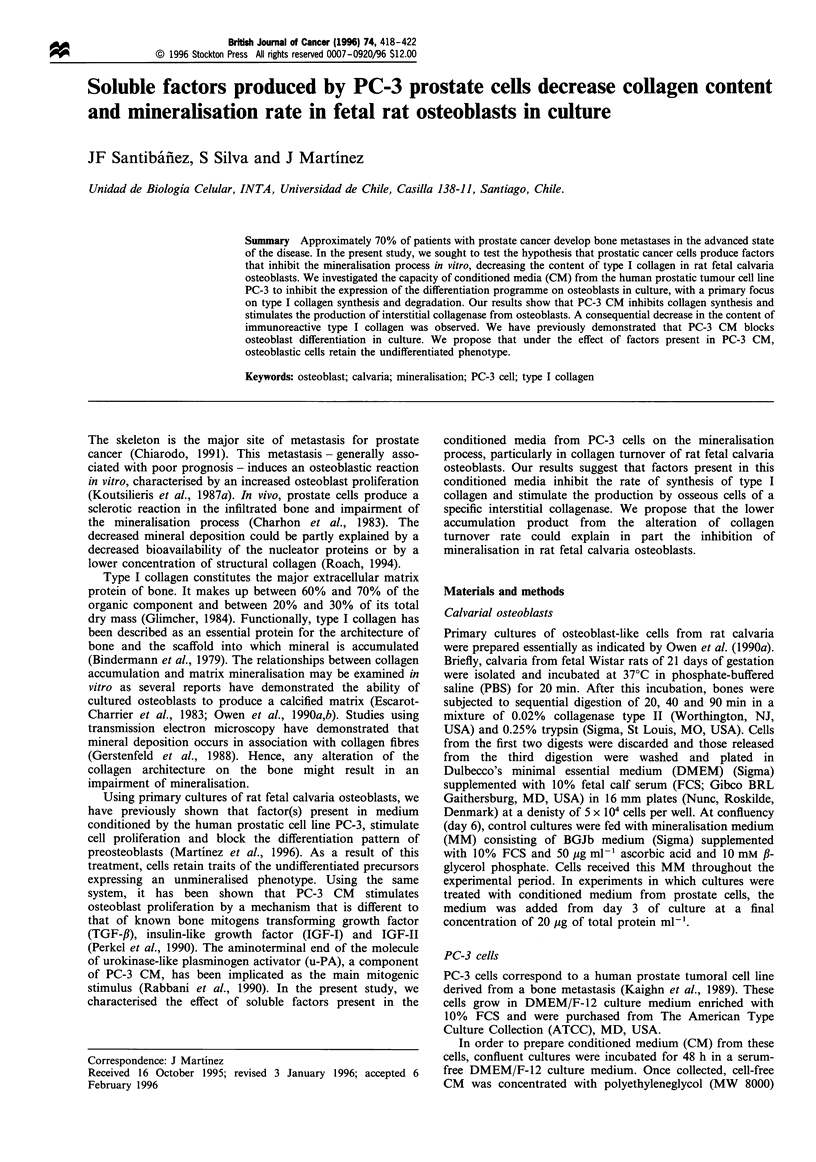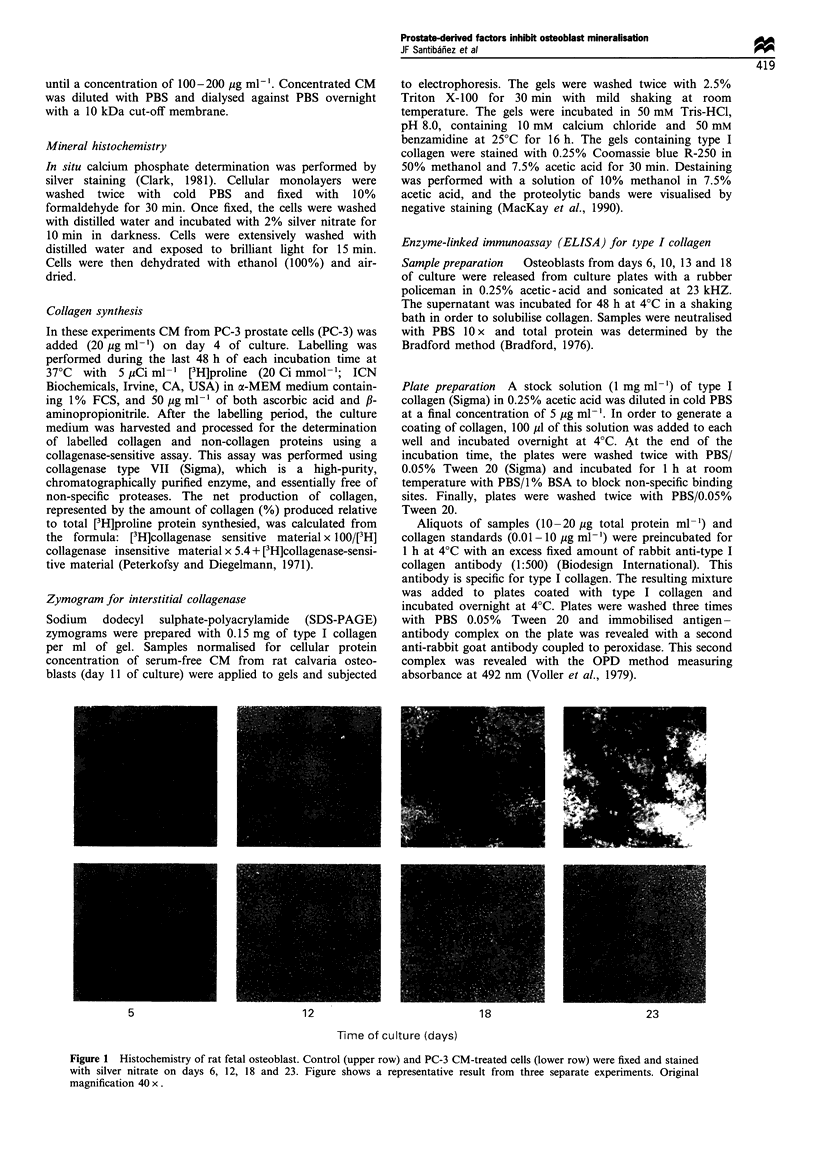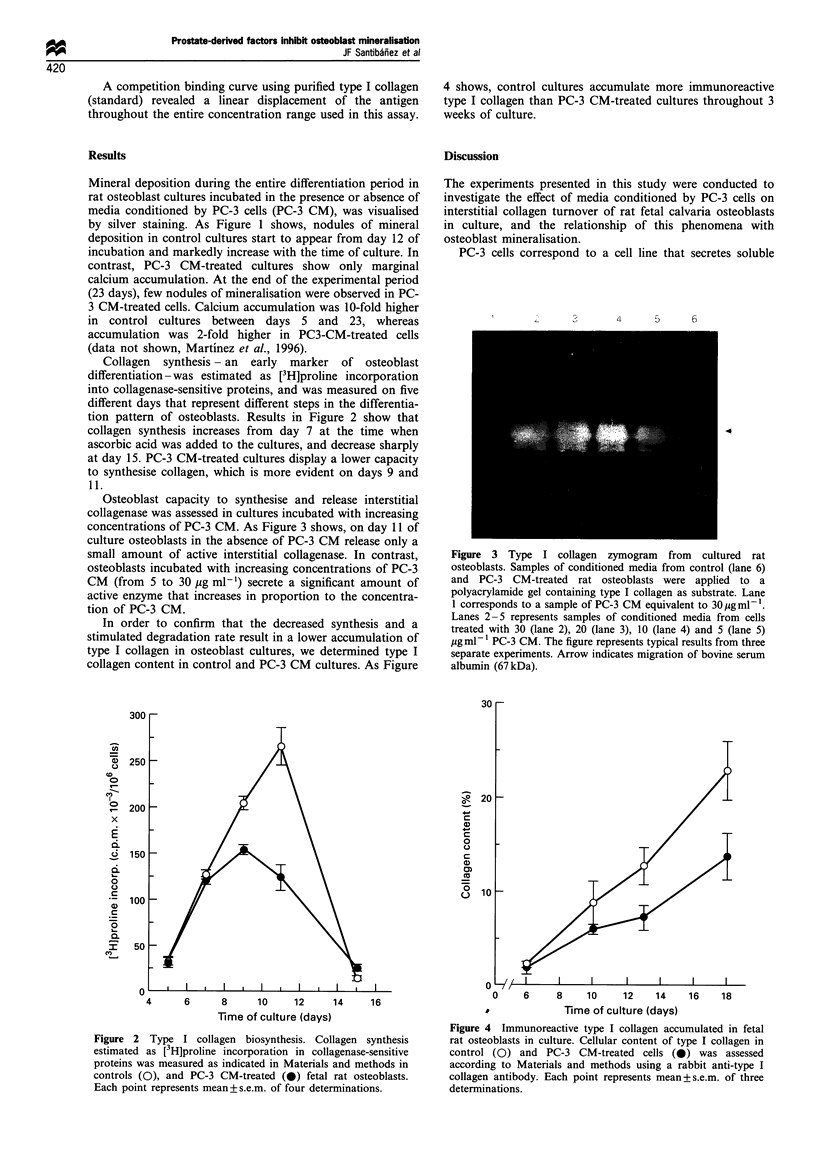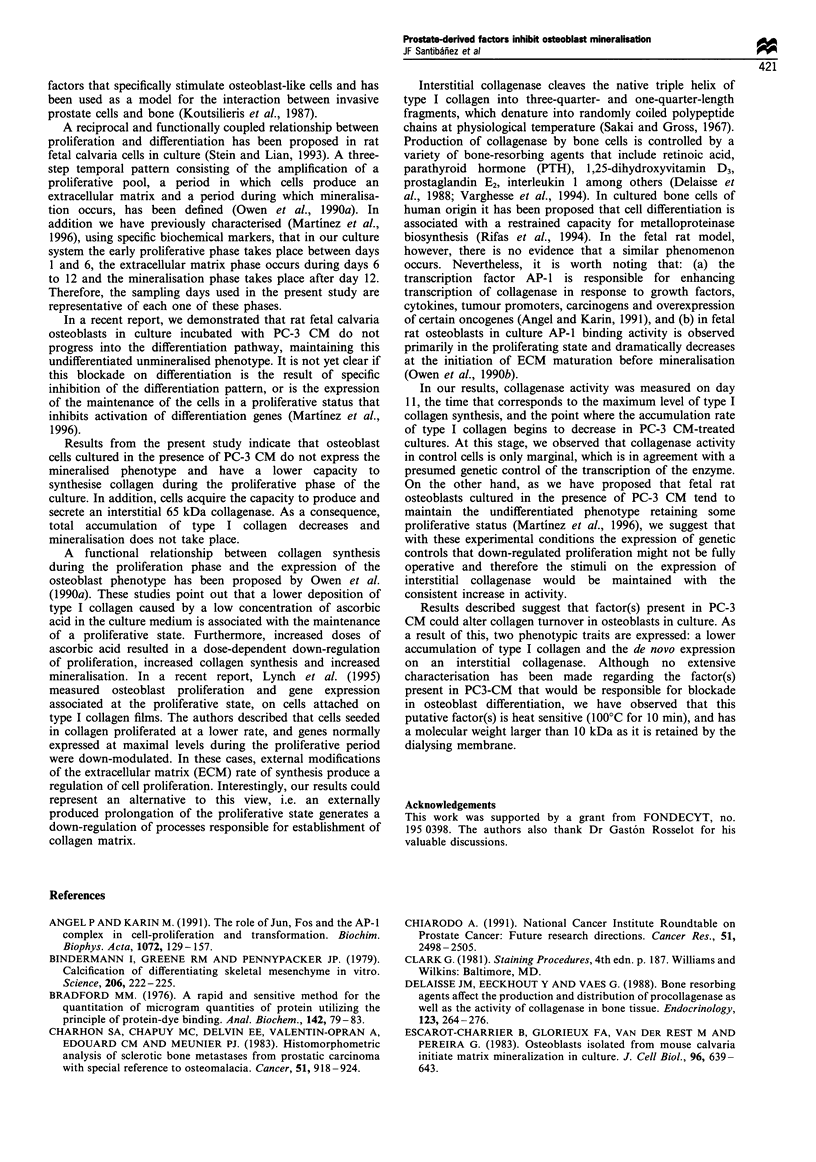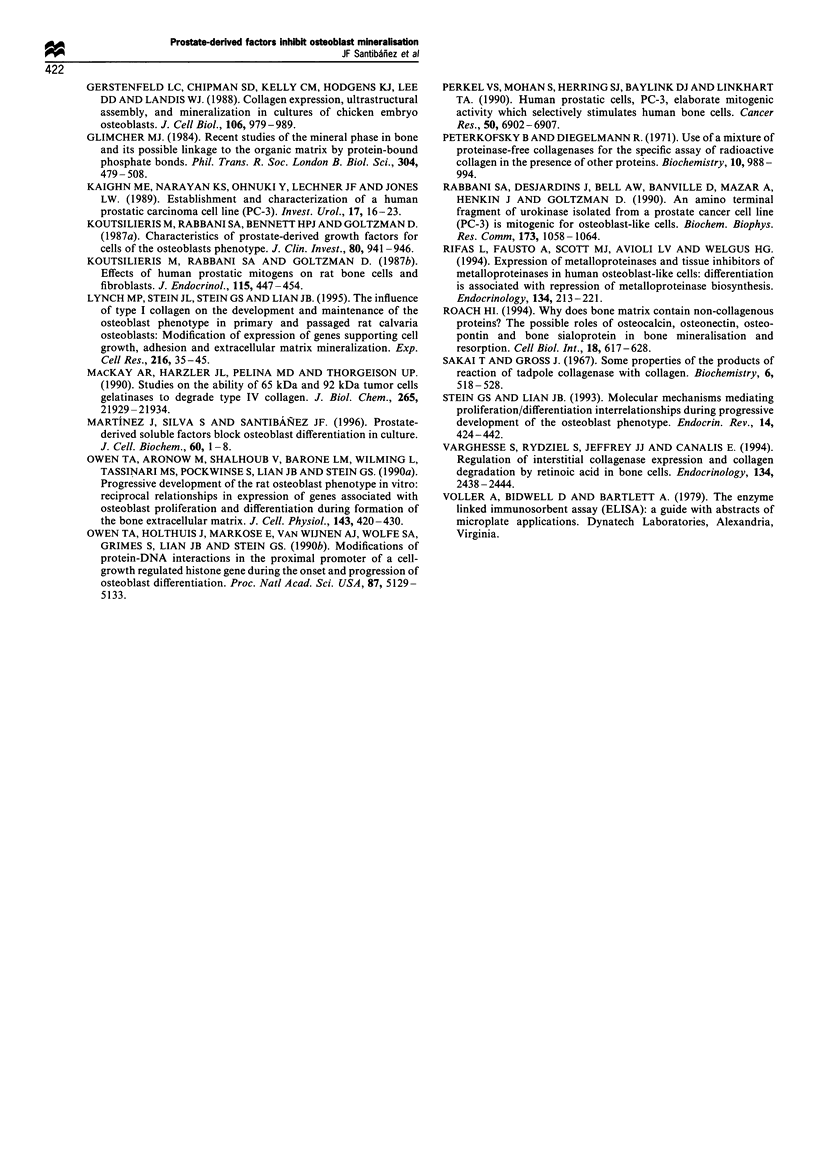# Soluble factors produced by PC-3 prostate cells decrease collagen content and mineralisation rate in fetal rat osteoblasts in culture.

**DOI:** 10.1038/bjc.1996.375

**Published:** 1996-08

**Authors:** J. F. Santibáñez, S. Silva, J. Martínez

**Affiliations:** Unidad de Biología Celular, INTA, Universidad de Chile, Santiago, Chile.

## Abstract

**Images:**


British Journal of Cancer (1996) 74, 418-422
© 1996 Stockton Press All rights reserved 0007-0920/96 $12.00

### Soluble factors produced by PC-3 prostate cells decrease collagen content and mineralisation rate in fetal rat osteoblasts in culture

JF Santibáñez, S Silva and J Martínez

*Unidad de Biología Celular, INTA, Universidad de Chile, Casilla 138-11, Santiago, Chile.*

**Summary** Approximately 70% of patients with prostate cancer develop bone metastases in the advanced state of the disease. In the present study, we sought to test the hypothesis that prostatic cancer cells produce factors that inhibit the mineralisation process *in vitro*, decreasing the content of type I collagen in rat fetal calvaria osteoblasts. We investigated the capacity of conditioned media (CM) from the human prostatic tumour cell line PC-3 to inhibit the expression of the differentiation programme on osteoblasts in culture, with a primary focus on type I collagen synthesis and degradation. Our results show that PC-3 CM inhibits collagen synthesis and stimulates the production of interstitial collagenase from osteoblasts. A consequential decrease in the content of immunoreactive type I collagen was observed. We have previously demonstrated that PC-3 CM blocks osteoblast differentiation in culture. We propose that under the effect of factors present in PC-3 CM, osteoblastic cells retain the undifferentiated phenotype.

**Keywords:** osteoblast; calvaria; mineralisation; PC-3 cell; type I collagen

The skeleton is the major site of metastasis for prostate cancer (Chiarodo, 1991). This metastasis – generally associated with poor prognosis – induces an osteoblastic reaction *in vitro*, characterised by an increased osteoblast proliferation (Koutsilieris *et al.*, 1987*a*). *In vivo*, prostate cells produce a sclerotic reaction in the infiltrated bone and impairment of the mineralisation process (Charhon *et al.*, 1983). The decreased mineral deposition could be partly explained by a decreased bioavailability of the nucleator proteins or by a lower concentration of structural collagen (Roach, 1994).

Type I collagen constitutes the major extracellular matrix protein of bone. It makes up between 60% and 70% of the organic component and between 20% and 30% of its total dry mass (Glimcher, 1984). Functionally, type I collagen has been described as an essential protein for the architecture of bone and the scaffold into which mineral is accumulated (Bindermann *et al.*, 1979). The relationships between collagen accumulation and matrix mineralisation may be examined *in vitro* as several reports have demonstrated the ability of cultured osteoblasts to produce a calcified matrix (Escarot-Charrier *et al.*, 1983; Owen *et al.*, 1990*a*,*b*). Studies using transmission electron microscopy have demonstrated that mineral deposition occurs in association with collagen fibres (Gerstenfeld *et al.*, 1988). Hence, any alteration of the collagen architecture on the bone might result in an impairment of mineralisation.

Using primary cultures of rat fetal calvaria osteoblasts, we have previously shown that factor(s) present in medium conditioned by the human prostatic cell line PC-3, stimulate cell proliferation and block the differentiation pattern of preosteoblasts (Martínez *et al.*, 1996). As a result of this treatment, cells retain traits of the undifferentiated precursors expressing an unmineralised phenotype. Using the same system, it has been shown that PC-3 CM stimulates osteoblast proliferation by a mechanism that is different to that of known bone mitogens transforming growth factor (TGF-$\beta$), insulin-like growth factor (IGF-I) and IGF-II (Perkel *et al.*, 1990). The aminoterminal end of the molecule of urokinase-like plasminogen activator (u-PA), a component of PC-3 CM, has been implicated as the main mitogenic stimulus (Rabbani *et al.*, 1990). In the present study, we characterised the effect of soluble factors present in the conditioned media from PC-3 cells on the mineralisation process, particularly in collagen turnover of rat fetal calvaria osteoblasts. Our results suggest that factors present in this conditioned media inhibit the rate of synthesis of type I collagen and stimulate the production by osseous cells of a specific interstitial collagenase. We propose that the lower accumulation product from the alteration of collagen turnover rate could explain in part the inhibition of mineralisation in rat fetal calvaria osteoblasts.

Correspondence: J Martínez
Received 16 October 1995; revised 3 January 1996; accepted 6 February 1996

### Materials and methods

### *Calvarial osteoblasts*

Primary cultures of osteoblast-like cells from rat calvaria were prepared essentially as indicated by Owen *et al.* (1990*a*). Briefly, calvaria from fetal Wistar rats of 21 days of gestation were isolated and incubated at 37°C in phosphate-buffered saline (PBS) for 20 min. After this incubation, bones were subjected to sequential digestion of 20, 40 and 90 min in a mixture of 0.02% collagenase type II (Worthington, NJ, USA) and 0.25% trypsin (Sigma, St Louis, MO, USA). Cells from the first two digests were discarded and those released from the third digestion were washed and plated in Dulbecco's minimal essential medium (DMEM) (Sigma) supplemented with 10% fetal calf serum (FCS; Gibco BRL Gaithersburg, MD, USA) in 16 mm plates (Nunc, Roskilde, Denmark) at a denisty of $5 \times 10^4$ cells per well. At confluency (day 6), control cultures were fed with mineralisation medium (MM) consisting of BGJb medium (Sigma) supplemented with 10% FCS and 50 $\mu g\ ml^{-1}$ ascorbic acid and 10 mM $\beta$-glycerol phosphate. Cells received this MM throughout the experimental period. In experiments in which cultures were treated with conditioned medium from prostate cells, the medium was added from day 3 of culture at a final concentration of 20 $\mu g$ of total protein $ml^{-1}$.

### *PC-3 cells*

PC-3 cells correspond to a human prostate tumoral cell line derived from a bone metastasis (Kaighn *et al.*, 1989). These cells grow in DMEM/F-12 culture medium enriched with 10% FCS and were purchased from The American Type Culture Collection (ATCC), MD, USA.

In order to prepare conditioned medium (CM) from these cells, confluent cultures were incubated for 48 h in a serum-free DMEM/F-12 culture medium. Once collected, cell-free CM was concentrated with polyethyleneglycol (MW 8000)

until a concentration of 100–200 μg $ml^{-1}$. Concentrated CM was diluted with PBS and dialysed against PBS overnight with a 10 kDa cut-off membrane.

*Mineral histochemistry*

*In situ* calcium phosphate determination was performed by silver staining (Clark, 1981). Cellular monolayers were washed twice with cold PBS and fixed with 10% formaldehyde for 30 min. Once fixed, the cells were washed with distilled water and incubated with 2% silver nitrate for 10 min in darkness. Cells were extensively washed with distilled water and exposed to brilliant light for 15 min. Cells were then dehydrated with ethanol (100%) and air-dried.

*Collagen synthesis*

In these experiments CM from PC-3 prostate cells (PC-3) was added (20 μg $ml^{-1}$) on day 4 of culture. Labelling was performed during the last 48 h of each incubation time at 37°C with 5 μCi $ml^{-1}$ [$^3$H]proline (20 Ci $mmol^{-1}$; ICN Biochemicals, Irvine, CA, USA) in α-MEM medium containing 1% FCS, and 50 μg $ml^{-1}$ of both ascorbic acid and β-aminopropionitrile. After the labelling period, the culture medium was harvested and processed for the determination of labelled collagen and non-collagen proteins using a collagenase-sensitive assay. This assay was performed using collagenase type VII (Sigma), which is a high-purity, chromatographically purified enzyme, and essentially free of non-specific proteases. The net production of collagen, represented by the amount of collagen (%) produced relative to total [$^3$H]proline protein synthesied, was calculated from the formula: [$^3$H]collagenase sensitive material × 100/[$^3$H] collagenase insensitive material × 5.4 + [$^3$H]collagenase-sensitive material (Peterkofsy and Diegelmann, 1971).

*Zymogram for interstitial collagenase*

Sodium dodecyl sulphate-polyacrylamide (SDS-PAGE) zymograms were prepared with 0.15 mg of type I collagen per ml of gel. Samples normalised for cellular protein concentration of serum-free CM from rat calvaria osteoblasts (day 11 of culture) were applied to gels and subjected to electrophoresis. The gels were washed twice with 2.5% Triton X-100 for 30 min with mild shaking at room temperature. The gels were incubated in 50 mM Tris-HCl, pH 8.0, containing 10 mM calcium chloride and 50 mM benzamidine at 25°C for 16 h. The gels containing type I collagen were stained with 0.25% Coomassie blue R-250 in 50% methanol and 7.5% acetic acid for 30 min. Destaining was performed with a solution of 10% methanol in 7.5% acetic acid, and the proteolytic bands were visualised by negative staining (MacKay *et al*., 1990).

*Enzyme-linked immunoassay (ELISA) for type I collagen*

*Sample preparation* Osteoblasts from days 6, 10, 13 and 18 of culture were released from culture plates with a rubber policeman in 0.25% acetic-acid and sonicated at 23 kHZ. The supernatant was incubated for 48 h at 4°C in a shaking bath in order to solubilise collagen. Samples were neutralised with PBS 10× and total protein was determined by the Bradford method (Bradford, 1976).

*Plate preparation* A stock solution (1 mg $ml^{-1}$) of type I collagen (Sigma) in 0.25% acetic acid was diluted in cold PBS at a final concentration of 5 μg $ml^{-1}$. In order to generate a coating of collagen, 100 μl of this solution was added to each well and incubated overnight at 4°C. At the end of the incubation time, the plates were washed twice with PBS/0.05% Tween 20 (Sigma) and incubated for 1 h at room temperature with PBS/1% BSA to block non-specific binding sites. Finally, plates were washed twice with PBS/0.05% Tween 20.

Aliquots of samples (10–20 μg total protein $ml^{-1}$) and collagen standards (0.01–10 μg $ml^{-1}$) were preincubated for 1 h at 4°C with an excess fixed amount of rabbit anti-type I collagen antibody (1:500) (Biodesign International). This antibody is specific for type I collagen. The resulting mixture was added to plates coated with type I collagen and incubated overnight at 4°C. Plates were washed three times with PBS 0.05% Tween 20 and immobilised antigen–antibody complex on the plate was revealed with a second anti-rabbit goat antibody coupled to peroxidase. This second complex was revealed with the OPD method measuring absorbance at 492 nm (Voller *et al*., 1979).

**Figure 1** Histochemistry of rat fetal osteoblast. Control (upper row) and PC-3 CM-treated cells (lower row) were fixed and stained with silver nitrate on days 6, 12, 18 and 23. Figure shows a representative result from three separate experiments. Original magnification 40×.

A competition binding curve using purified type I collagen (standard) revealed a linear displacement of the antigen throughout the entire concentration range used in this assay.

### Results

Mineral deposition during the entire differentiation period in rat osteoblast cultures incubated in the presence or absence of media conditioned by PC-3 cells (PC-3 CM), was visualised by silver staining. As Figure 1 shows, nodules of mineral deposition in control cultures start to appear from day 12 of incubation and markedly increase with the time of culture. In contrast, PC-3 CM-treated cultures show only marginal calcium accumulation. At the end of the experimental period (23 days), few nodules of mineralisation were observed in PC-3 CM-treated cells. Calcium accumulation was 10-fold higher in control cultures between days 5 and 23, whereas accumulation was 2-fold higher in PC3-CM-treated cells (data not shown, Martínez *et al.*, 1996).

Collagen synthesis – an early marker of osteoblast differentiation – was estimated as [$^3$H]proline incorporation into collagenase-sensitive proteins, and was measured on five different days that represent different steps in the differentiation pattern of osteoblasts. Results in Figure 2 show that collagen synthesis increases from day 7 at the time when ascorbic acid was added to the cultures, and decrease sharply at day 15. PC-3 CM-treated cultures display a lower capacity to synthesise collagen, which is more evident on days 9 and 11.

Osteoblast capacity to synthesise and release interstitial collagenase was assessed in cultures incubated with increasing concentrations of PC-3 CM. As Figure 3 shows, on day 11 of culture osteoblasts in the absence of PC-3 CM release only a small amount of active interstitial collagenase. In contrast, osteoblasts incubated with increasing concentrations of PC-3 CM (from 5 to 30 $\mu$g ml$^{-1}$) secrete a significant amount of active enzyme that increases in proportion to the concentration of PC-3 CM.

In order to confirm that the decreased synthesis and a stimulated degradation rate result in a lower accumulation of type I collagen in osteoblast cultures, we determined type I collagen content in control and PC-3 CM cultures. As Figure 4 shows, control cultures accumulate more immunoreactive type I collagen than PC-3 CM-treated cultures throughout 3 weeks of culture.

### Discussion

The experiments presented in this study were conducted to investigate the effect of media conditioned by PC-3 cells on interstitial collagen turnover of rat fetal calvaria osteoblasts in culture, and the relationship of this phenomena with osteoblast mineralisation.

PC-3 cells correspond to a cell line that secretes soluble

**Figure 3** Type I collagen zymogram from cultured rat osteoblasts. Samples of conditioned media from control (lane 6) and PC-3 CM-treated rat osteoblasts were applied to a polyacrylamide gel containing type I collagen as substrate. Lane 1 corresponds to a sample of PC-3 CM equivalent to 30 $\mu$g ml$^{-1}$. Lanes 2–5 represents samples of conditioned media from cells treated with 30 (lane 2), 20 (lane 3), 10 (lane 4) and 5 (lane 5) $\mu$g ml$^{-1}$ PC-3 CM. The figure represents typical results from three separate experiments. Arrow indicates migration of bovine serum albumin (67 kDa).

**Figure 2** Type I collagen biosynthesis. Collagen synthesis estimated as [$^3$H]proline incorporation in collagenase-sensitive proteins was measured as indicated in Materials and methods in controls (○), and PC-3 CM-treated (●) fetal rat osteoblasts. Each point represents mean ± s.e.m. of four determinations.

**Figure 4** Immunoreactive type I collagen accumulated in fetal rat osteoblasts in culture. Cellular content of type I collagen in control (○) and PC-3 CM-treated cells (●) was assessed according to Materials and methods using a rabbit anti-type I collagen antibody. Each point represents mean ± s.e.m. of three determinations.

factors that specifically stimulate osteoblast-like cells and has been used as a model for the interaction between invasive prostate cells and bone (Koutsilieris *et al.*, 1987).

A reciprocal and functionally coupled relationship between proliferation and differentiation has been proposed in rat fetal calvaria cells in culture (Stein and Lian, 1993). A three-step temporal pattern consisting of the amplification of a proliferative pool, a period in which cells produce an extracellular matrix and a period during which mineralisation occurs, has been defined (Owen *et al.*, 1990*a*). In addition we have previously characterised (Martínez *et al.*, 1996), using specific biochemical markers, that in our culture system the early proliferative phase takes place between days 1 and 6, the extracellular matrix phase occurs during days 6 to 12 and the mineralisation phase takes place after day 12. Therefore, the sampling days used in the present study are representative of each one of these phases.

In a recent report, we demonstrated that rat fetal calvaria osteoblasts in culture incubated with PC-3 CM do not progress into the differentiation pathway, maintaining this undifferentiated unmineralised phenotype. It is not yet clear if this blockade on differentiation is the result of specific inhibition of the differentiation pattern, or is the expression of the maintenance of the cells in a proliferative status that inhibits activation of differentiation genes (Martínez *et al.*, 1996).

Results from the present study indicate that osteoblast cells cultured in the presence of PC-3 CM do not express the mineralised phenotype and have a lower capacity to synthesise collagen during the proliferative phase of the culture. In addition, cells acquire the capacity to produce and secrete an interstitial 65 kDa collagenase. As a consequence, total accumulation of type I collagen decreases and mineralisation does not take place.

A functional relationship between collagen synthesis during the proliferation phase and the expression of the osteoblast phenotype has been proposed by Owen *et al.* (1990*a*). These studies point out that a lower deposition of type I collagen caused by a low concentration of ascorbic acid in the culture medium is associated with the maintenance of a proliferative state. Furthermore, increased doses of ascorbic acid resulted in a dose-dependent down-regulation of proliferation, increased collagen synthesis and increased mineralisation. In a recent report, Lynch *et al.* (1995) measured osteoblast proliferation and gene expression associated at the proliferative state, on cells attached on type I collagen films. The authors described that cells seeded in collagen proliferated at a lower rate, and genes normally expressed at maximal levels during the proliferative period were down-modulated. In these cases, external modifications of the extracellular matrix (ECM) rate of synthesis produce a regulation of cell proliferation. Interestingly, our results could represent an alternative to this view, i.e. an externally produced prolongation of the proliferative state generates a down-regulation of processes responsible for establishment of collagen matrix.

Interstitial collagenase cleaves the native triple helix of type I collagen into three-quarter- and one-quarter-length fragments, which denature into randomly coiled polypeptide chains at physiological temperature (Sakai and Gross, 1967). Production of collagenase by bone cells is controlled by a variety of bone-resorbing agents that include retinoic acid, parathyroid hormone (PTH), 1,25-dihydroxyvitamin $D_3$, prostaglandin $E_2$, interleukin 1 among others (Delaisse *et al.*, 1988; Varghesse *et al.*, 1994). In cultured bone cells of human origin it has been proposed that cell differentiation is associated with a restrained capacity for metalloproteinase biosynthesis (Rifas *et al.*, 1994). In the fetal rat model, however, there is no evidence that a similar phenomenon occurs. Nevertheless, it is worth noting that: (a) the transcription factor AP-1 is responsible for enhancing transcription of collagenase in response to growth factors, cytokines, tumour promoters, carcinogens and overexpression of certain oncogenes (Angel and Karin, 1991), and (b) in fetal rat osteoblasts in culture AP-1 binding activity is observed primarily in the proliferating state and dramatically decreases at the initiation of ECM maturation before mineralisation (Owen *et al.*, 1990*b*).

In our results, collagenase activity was measured on day 11, the time that corresponds to the maximum level of type I collagen synthesis, and the point where the accumulation rate of type I collagen begins to decrease in PC-3 CM-treated cultures. At this stage, we observed that collagenase activity in control cells is only marginal, which is in agreement with a presumed genetic control of the transcription of the enzyme. On the other hand, as we have proposed that fetal rat osteoblasts cultured in the presence of PC-3 CM tend to maintain the undifferentiated phenotype retaining some proliferative status (Martínez *et al.*, 1996), we suggest that with these experimental conditions the expression of genetic controls that down-regulated proliferation might not be fully operative and therefore the stimuli on the expression of interstitial collagenase would be maintained with the consistent increase in activity.

Results described suggest that factor(s) present in PC-3 CM could alter collagen turnover in osteoblasts in culture. As a result of this, two phenotypic traits are expressed: a lower accumulation of type I collagen and the *de novo* expression on an interstitial collagenase. Although no extensive characterisation has been made regarding the factor(s) present in PC3-CM that would be responsible for blockade in osteoblast differentiation, we have observed that this putative factor(s) is heat sensitive (100°C for 10 min), and has a molecular weight larger than 10 kDa as it is retained by the dialysing membrane.

**Acknowledgements**
This work was supported by a grant from FONDECYT, no. 195 0398. The authors also thank Dr Gastón Rosselot for his valuable discussions.

### References

ANGEL P AND KARIN M. (1991). The role of Jun, Fos and the AP-1 complex in cell-proliferation and transformation. *Biochim. Biophys. Acta*, **1072,** 129–157.

BINDERMANN I, GREENE RM AND PENNYPACKER JP. (1979). Calcification of differentiating skeletal mesenchyme in vitro. *Science*, **206,** 222–225.

BRADFORD MM. (1976). A rapid and sensitive method for the quantitation of microgram quantities of protein utilizing the principle of protein-dye binding. *Anal. Biochem.*, **142,** 79–83.

CHARHON SA, CHAPUY MC, DELVIN EE, VALENTIN-OPRAN A, EDOUARD CM AND MEUNIER PJ. (1983). Histomorphometric analysis of sclerotic bone metastases from prostatic carcinoma with special reference to osteomalacia. *Cancer*, **51,** 918–924.

CHIARODO A. (1991). National Cancer Institute Roundtable on Prostate Cancer: Future research directions. *Cancer Res.*, **51,** 2498–2505.

CLARK G. (1981). *Staining Procedures*, 4th edn. p. 187. Williams and Wilkins: Baltimore, MD.

DELAISSE JM, EECKHOUT Y AND VAES G. (1988). Bone resorbing agents affect the production and distribution of procollagenase as well as the activity of collagenase in bone tissue. *Endocrinology*, **123,** 264–276.

ESCAROT-CHARRIER B, GLORIEUX FA, VAN DER REST M AND PEREIRA G. (1983). Osteoblasts isolated from mouse calvaria initiate matrix mineralization in culture. *J. Cell Biol.*, **96,** 639–643.

GERSTENFELD LC, CHIPMAN SD, KELLY CM, HODGENS KJ, LEE DD AND LANDIS WJ. (1988). Collagen expression, ultrastructural assembly, and mineralization in cultures of chicken embryo osteoblasts. *J. Cell Biol.*, **106**, 979–989.

GLIMCHER MJ. (1984). Recent studies of the mineral phase in bone and its possible linkage to the organic matrix by protein-bound phosphate bonds. *Phil. Trans. R. Soc. London B. Biol. Sci.*, **304**, 479–508.

KAIGHN ME, NARAYAN KS, OHNUKI Y, LECHNER JF AND JONES LW. (1989). Establishment and characterization of a human prostatic carcinoma cell line (PC-3). *Invest. Urol.*, **17**, 16–23.

KOUTSILIERIS M, RABBANI SA, BENNETT HPJ AND GOLTZMAN D. (1987*a*). Characteristics of prostate-derived growth factors for cells of the osteoblasts phenotype. *J. Clin. Invest.*, **80**, 941–946.

KOUTSILIERIS M, RABBANI SA AND GOLTZMAN D. (1987*b*). Effects of human prostatic mitogens on rat bone cells and fibroblasts. *J. Endocrinol.*, **115**, 447–454.

LYNCH MP, STEIN JL, STEIN GS AND LIAN JB. (1995). The influence of type I collagen on the development and maintenance of the osteoblast phenotype in primary and passaged rat calvaria osteoblasts: Modification of expression of genes supporting cell growth, adhesion and extracellular matrix mineralization. *Exp. Cell Res.*, **216**, 35–45.

MACKAY AR, HARZLER JL, PELINA MD AND THORGEISON UP. (1990). Studies on the ability of 65 kDa and 92 kDa tumor cells gelatinases to degrade type IV collagen. *J. Biol. Chem.*, **265**, 21929–21934.

MARTÍNEZ J, SILVA S AND SANTIBÁÑEZ JF. (1996). Prostate-derived soluble factors block osteoblast differentiation in culture. *J. Cell. Biochem.*, **60**, 1–8.

OWEN TA, ARONOW M, SHALHOUB V, BARONE LM, WILMING L, TASSINARI MS, POCKWINSE S, LIAN JB AND STEIN GS. (1990*a*). Progressive development of the rat osteoblast phenotype in vitro: reciprocal relationships in expression of genes associated with osteoblast proliferation and differentiation during formation of the bone extracellular matrix. *J. Cell. Physiol.*, **143**, 420–430.

OWEN TA, HOLTHUIS J, MARKOSE E, VAN WIJNEN AJ, WOLFE SA, GRIMES S, LIAN JB AND STEIN GS. (1990*b*). Modifications of protein-DNA interactions in the proximal promoter of a cell-growth regulated histone gene during the onset and progression of osteoblast differentiation. *Proc. Natl Acad. Sci. USA*, **87**, 5129–5133.

PERKEL VS, MOHAN S, HERRING SJ, BAYLINK DJ AND LINKHART TA. (1990). Human prostatic cells, PC-3, elaborate mitogenic activity which selectively stimulates human bone cells. *Cancer Res.*, **50**, 6902–6907.

PETERKOFSKY B AND DIEGELMANN R. (1971). Use of a mixture of proteinase-free collagenases for the specific assay of radioactive collagen in the presence of other proteins. *Biochemistry*, **10**, 988–994.

RABBANI SA, DESJARDINS J, BELL AW, BANVILLE D, MAZAR A, HENKIN J AND GOLTZMAN D. (1990). An amino terminal fragment of urokinase isolated from a prostate cancer cell line (PC-3) is mitogenic for osteoblast-like cells. *Biochem. Biophys. Res. Comm*, **173**, 1058–1064.

RIFAS L, FAUSTO A, SCOTT MJ, AVIOLI LV AND WELGUS HG. (1994). Expression of metalloproteinases and tissue inhibitors of metalloproteinases in human osteoblast-like cells: differentiation is associated with repression of metalloproteinase biosynthesis. *Endocrinology*, **134**, 213–221.

ROACH HI. (1994). Why does bone matrix contain non-collagenous proteins? The possible roles of osteocalcin, osteonectin, osteopontin and bone sialoprotein in bone mineralisation and resorption. *Cell Biol. Int.*, **18**, 617–628.

SAKAI T AND GROSS J. (1967). Some properties of the products of reaction of tadpole collagenase with collagen. *Biochemistry*, **6**, 518–528.

STEIN GS AND LIAN JB. (1993). Molecular mechanisms mediating proliferation/differentiation interrelationships during progressive development of the osteoblast phenotype. *Endocrin. Rev.*, **14**, 424–442.

VARGHESSE S, RYDZIEL S, JEFFREY JJ AND CANALIS E. (1994). Regulation of interstitial collagenase expression and collagen degradation by retinoic acid in bone cells. *Endocrinology*, **134**, 2438–2444.

VOLLER A, BIDWELL D AND BARTLETT A. (1979). The enzyme linked immunosorbent assay (ELISA): a guide with abstracts of microplate applications. Dynatech Laboratories, Alexandria, Virginia.